# *Thermus* and the Pink Discoloration Defect in Cheese

**DOI:** 10.1128/mSystems.00023-16

**Published:** 2016-06-14

**Authors:** Lisa Quigley, Daniel J. O’Sullivan, David Daly, Orla O’Sullivan, Zuzana Burdikova, Rostislav Vana, Tom P. Beresford, R. Paul Ross, Gerald F. Fitzgerald, Paul L. H. McSweeney, Linda Giblin, Jeremiah J. Sheehan, Paul D. Cotter

**Affiliations:** a Teagasc Food Research Centre, Moorepark, Fermoy, Cork, Ireland; b School of Microbiology, University College Cork, Cork, Ireland; c School of Food and Nutritional Sciences, University College Cork, Cork, Ireland; d Tescan Orsay Holding, Brno, Czech Republic; e APC Microbiome Institute, Cork, Ireland; f College of Science, Engineering and Food Science, University College Cork, Cork, Ireland; University of California, San Diego

**Keywords:** carotenoid, cheese, microbiota, pinking, *Thermus*

## Abstract

A DNA sequencing-based strategy was applied to study the microbiology of Continental-type cheeses with a pink discoloration defect. The basis for this phenomenon has remained elusive, despite decades of research. The bacterial composition of cheese containing the defect was compared to that of control cheese using 16S rRNA gene and shotgun metagenomic sequencing as well as quantitative PCR (qPCR). Throughout, it was apparent that *Thermus*, a carotenoid-producing genus, was present at higher levels in defect-associated cheeses than in control cheeses. Prompted by this finding and data confirming the pink discoloration to be associated with the presence of a carotenoid, a culture-based approach was employed, and *Thermus thermophilus* was successfully cultured from defect-containing cheeses. The link between *Thermus* and the pinking phenomenon was then established through the cheese defect equivalent of Koch’s postulates when the defect was recreated by the reintroduction of a *T. thermophilus* isolate to a test cheese during the manufacturing process.

**IMPORTANCE** Pink discoloration in cheese is a defect affecting many cheeses throughout the world, leading to significant financial loss for the dairy industry. Despite decades of research, the cause of this defect has remained elusive. The advent of high-throughput, next-generation sequencing has revolutionized the field of food microbiology and, with respect to this study, provided a means of testing a possible microbial basis for this defect. In this study, a combined 16S rRNA, whole-genome sequencing, and quantitative PCR approach was taken. This resulted in the identification of *Thermus*, a carotenoid-producing thermophile, in defect-associated cheeses and the recreation of the problem in cheeses to which *Thermus* was added. This finding has the potential to lead to new strategies to eliminate this defect, and our method represents an approach that can be employed to investigate the role of microbes in other food defects of unknown origin.

## Abstract (precis)

Pink discoloration in cheese is a defect affecting many cheeses throughout the world, leading to significant financial loss for the dairy industry. Despite decades of research, the cause of this defect has remained elusive. The advent of high-throughput, next-generation sequencing has revolutionized the field of food microbiology and, with respect to this study, provided a means of testing a possible microbial basis for this defect. In this study, a combined 16S rRNA, whole-genome sequencing, and quantitative PCR approach was taken. This resulted in the identification of *Thermus*, a carotenoid-producing thermophile, in defect-associated cheeses and the recreation of the problem in cheeses to which *Thermus* was added. This finding has the potential to lead to new strategies to eliminate this defect, and our method represents an approach that can be employed to investigate the role of microbes in other food defects of unknown origin.

## INTRODUCTION

The pink discoloration defect is a problem that affects the cheese industry worldwide (1). Despite first being noted in the scientific literature in 1933 (2) and being the subject of extensive research, the basis for this phenomenon has remained elusive. It particularly impacts a range of ripened cheeses, including Swiss, Cheddar, and Italian-type cheeses (3–8), and results in the downgrading or rejection of cheese and a consequential economic loss (1). The defect can manifest in a number of ways, depending on the cheese type: at the surface of the cheese block (in patches or over the entire surface), as a uniform pink border occurring below the external surfaces of the cheese block, conferring a pink ring appearance, or distributed sporadically within the cheese block (1). Pink discoloration affects cheeses both with and without additional colorants. In cheeses with colorants, such as annatto, pink discoloration is thought to be a result of factors (oxidation, precipitation, temperature, and photooxidation) affecting the constituents of the colorant itself (1, 5). Contrastingly, in cheeses without colorants, the cause of this defect is unknown. There have been suggestions that it is due to physicochemical factors (Maillard browning) (5, 9–11), while some have proposed a microbial basis (8, 12). In the latter case, it has been claimed that cheeses containing specific starter cultures, and thermophilic strains of lactobacilli and propionic acid bacteria (PAB) in particular, are more likely to have a pink discoloration (6, 8, 13), but this has been the subject of much debate, and no clear consensus has been achieved.

High-throughput DNA sequencing technologies have provided detailed insight into the microbial compositions of a wide variety of different ecosystems (14), as well as of a selection of food-associated niches (15), including, more recently, dairy-based foods (16–19), revealing novel, albeit in many cases descriptive, findings. Here we employ a combination of 16S rRNA gene and shotgun metagenomic sequencing, quantitative PCR (qPCR), culture-based microbiology, and cheese manufacturing to identify the microbial component responsible for the pink discoloration phenomenon.

## RESULTS

### Compositional sequencing reveals high proportions of the genus *Thermus* in cheeses with a pink defect.

**FIG 1:**
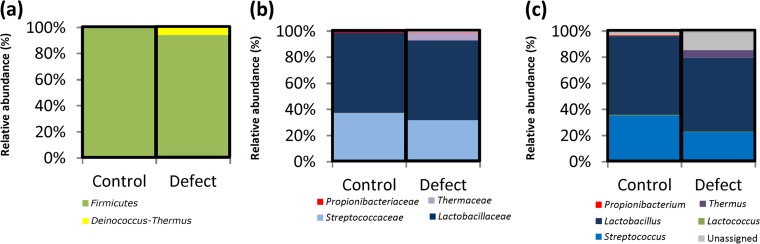
Bacterial compositions of defect-associated and control cheeses as determined by 16S rRNA gene sequencing. 16S rRNA gene sequences were assigned according to MEGAN using the Silva database at the phylum (a), family (b), and genus (c) levels in Continental-type cheese affected by the pink discoloration defect and in corresponding control cheeses (*n =* 18).

Compositional (V4-V5 region of 16S rRNA genes) sequencing was performed on DNA extracted from control (*n =* 9) and pink-defect (*n =* 9) samples of a commercially produced Continental-type cheese. Sequencing coverage was satisfactory for all samples (see Fig. S1 in the supplemental material). Phylogenetic analysis established that the sequence reads corresponded to five different bacterial phyla (Fig. 1a), i.e., *Firmicutes*, *Proteobacteria*, *Bacteroides*, *Actinobacteria*, and *Deinococcus*-*Thermus*. *Firmicutes* and *Deinococcus*-*Thermus* dominated, with less than 1% of assigned reads corresponding to other phyla. The proportions of *Firmicutes* present did not differ between control and pink-defect samples. Reads corresponding to the phylum *Deinococcus*-*Thermus* were detected in defect-associated samples only (6%). When reads were assigned at the family level, 11 families were identified (Fig. 1b). All reads from the phylum *Deinococcus*-*Thermus* were assigned to the family *Thermaceae*, and again, this was the only taxon for which significant differences were observed (i.e., organisms occurred in 6% and 0% of defect-associated and control samples, respectively). When these reads were assigned at the genus level, 10 genera were identified (Fig. 1c; Table S1). Reads corresponding to *Deinococcus*-*Thermus* and *Thermaceae* were assigned to the genus *Thermus*, and again, this was the only taxonomic group for which there were significant differences (*P* = 0.002).

10.1128/mSystems.00023-16.1 Figure S1  16S rRNA sequencing read analysis. The number of 16S rRNA reads per cheese was ≥3,500 (the average number of reads per sample was 3,960). A rarefaction curve of α-diversity values, represented by Shannon indices, for all samples sequenced confirmed that satisfactory coverage was achieved. Sequence data have been uploaded to the European Nucleotide Archive (ENA) under accession number PRJEB6952. Download Figure S1, DOCX file, 0.1 MB. Copyright © 2016 Quigley et al. 2016 Quigley et al. This content is distributed under the terms of the Creative Commons Attribution 4.0 International license.

10.1128/mSystems.00023-16.6 Table S1  List of 16S rRNA reads assigned at the genus level to control and defect-associated cheeses. Download Table S1, DOCX file, 0.01 MB. Copyright © 2016 Quigley et al. 2016 Quigley et al. This content is distributed under the terms of the Creative Commons Attribution 4.0 International license.

### Shotgun metagenomic sequencing provides further insight into the *Thermus* organisms and associated pathways that are prevalent in pink-defect cheeses.

**FIG 2:**
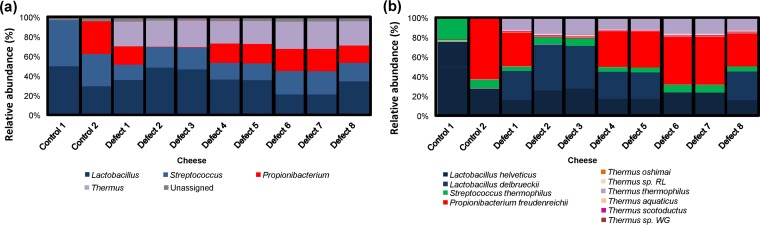
Bacterial compositions of defect-associated and control cheeses as determined by shotgun metagenomic sequencing. Sequences were assigned according to MEGAN at the genus (a) and species (b) levels for cheeses affected by the pink discoloration defect and corresponding control cheeses (*n =* 10). At the species level, unassigned populations were omitted.

Ten additional samples of Continental-type cheeses, i.e., two control cheeses and eight pink-defect cheeses, were selected for shotgun metagenomic sequencing. A total of 231,401,379 reads (single reads of 150 bp) after quality filtering was performed were obtained and assembled (an average of 89.1% reads per sample were assembled). Phylogenetic analysis revealed the presence of bacteria corresponding to three phyla, *Firmicutes*, *Actinobacteria*, and *Deinococcus*-*Thermus* (see Fig. S2 in the supplemental material). *Firmicutes* were again a dominant component across all samples, but unlike with previous compositional data, *Actinobacteria* were also present in high proportions across many samples (reflecting a deficiency in the binding of the 16S rRNA gene primers used for compositional sequencing to *Propionibacterium*) (Table S2). *Deinococcus-Thermus* populations were again present in defect-associated samples only (24 to 28% of assigned reads). These organisms corresponded primarily to *Thermus* at the genus level, though subdominant populations corresponding to *Meiothermus* and *Deinococcus* were also detected (Fig. 2a; Table S3a). Shotgun analysis also allowed assignment at the species level, which revealed consistently high levels of *Lactobacillus helveticus*, *Streptococcus thermophilus*, and, in many cases, *Propionibacterium freudenreichii* (Fig. 2b). All three are starters used in the manufacture of this Continental-type cheese. Several members of the *Thermus* genus were present, including *T. thermophilus*, *T. aquaticus*, *T. scotoductus*, *T. oshimai*, *Thermus* sp. strain RL, and *Thermus* sp. strain WG. Of these, *T. thermophilus* dominated, corresponding to 5.9 to 7.03% of assigned reads (Fig. 2b; Table S4).

10.1128/mSystems.00023-16.2 Figure S2  Bacterial compositions of defect-associated and control cheeses as determined by shotgun metagenomic sequencing**.** Sequences were assigned according to MEGAN at the phylum level for cheese affected by the pink discoloration defect and corresponding control cheeses. Download Figure S2, DOCX file, 0.1 MB. Copyright © 2016 Quigley et al. 2016 Quigley et al. This content is distributed under the terms of the Creative Commons Attribution 4.0 International license.

10.1128/mSystems.00023-16.7 Table S2  BLAST analysis of degenerate primers used in 454 compositional sequencing against the *P. freudenreichii* subsp. *shermanii* genome. The absence of similarity to the forward primer explains the differences between *Propionibacterium* populations detected via compositional and shotgun sequencing. Download Table S2, DOCX file, 0.01 MB. Copyright © 2016 Quigley et al. 2016 Quigley et al. This content is distributed under the terms of the Creative Commons Attribution 4.0 International license.

10.1128/mSystems.00023-16.8 Table S3  Shotgun metagenomic sequences per sample assigned at the genus level (a) and at the species level (b) to control and defect-associated cheeses (as a percentage of those assigned). Download Table S3, DOCX file, 0.02 MB. Copyright © 2016 Quigley et al. 2016 Quigley et al. This content is distributed under the terms of the Creative Commons Attribution 4.0 International license.

10.1128/mSystems.00023-16.9 Table S4  Assessments carried out at different stages of manufacture and ripening. Download Table S4, DOCX file, 0.01 MB. Copyright © 2016 Quigley et al. 2016 Quigley et al. This content is distributed under the terms of the Creative Commons Attribution 4.0 International license.

10.1128/mSystems.00023-16.10 Table S5  Compositions of cheeses at 11 days postmanufacture. Download Table S5, DOCX file, 0.01 MB. Copyright © 2016 Quigley et al. 2016 Quigley et al. This content is distributed under the terms of the Creative Commons Attribution 4.0 International license.

**FIG 3:**
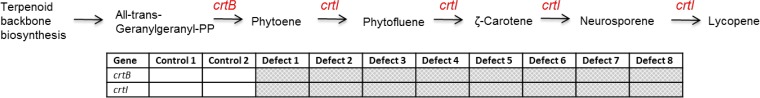
Carotenoid biosynthesis pathway genes detected in cheeses exhibiting a pinking defect. The detection of reads corresponding to the *crtB* and *crtI* genes in specific cheeses is indicated by the shaded boxes.

**FIG 4:**
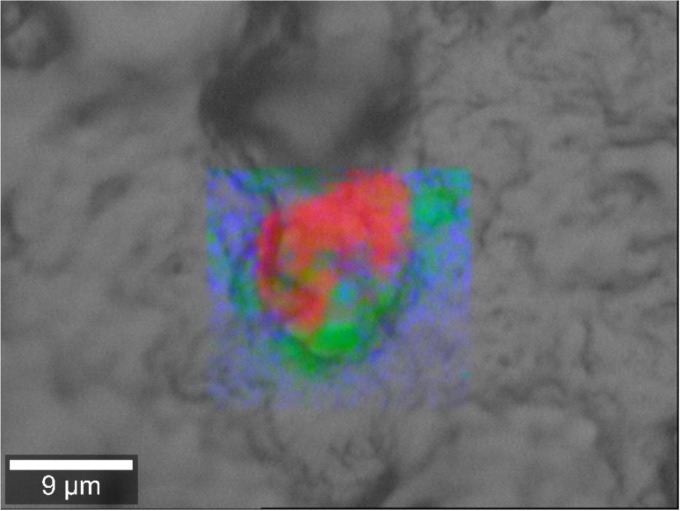
Overlay of intensity images of the studied cheese matrix (gray) and the maps of the chemical composition obtained from local Raman spectral analysis of a pink discoloration region of a defect-associated cheese: red, carotenoid (lycopane); blue, proteins; green, lipids.

Functional analysis of this sequence data was performed, with 95,827 genes being assigned across all samples (an overview of the KEGG pathways present can be found in Fig. S3 in the supplemental material). Unsurprisingly, given the presence of reads corresponding to *Thermus* in the defect-associated samples exclusively, it was noted that genes responsible for the production of carotenoids were identified in defect-associated samples only (Fig. 3). Notably, Raman spectra of samples from regions of pink discoloration within pink-defect cheeses (Fig. S4) revealed a peak at 1,456 cm^−1^, characteristic of lycopane (perhydro-transformed carotenoid from lycopene) (20), and is absent from nonpink regions from the same cheese. The pink layer also shows very strong peaks at 877 cm^−1^ and 990 cm^−1^, which are consistent with v1(PO_4_^3−^) of a phosphate salt. The localized distribution of prominent Raman peaks at 990 cm^−1^ and 1,456 cm^−1^ (carotenoid-phosphate salt; corresponding to red), 1,441 cm^−1^ and 2,840 to 2,945 cm^−1^ (proteins; corresponding to blue), and 3,060 cm^−1^ (lipids; corresponding to green) is shown in Fig. 4.

10.1128/mSystems.00023-16.3 Figure S3  Breakdown of KEGG pathways present. Bar graph data are percentages of assigned reads. Download Figure S3, DOCX file, 0.2 MB. Copyright © 2016 Quigley et al. 2016 Quigley et al. This content is distributed under the terms of the Creative Commons Attribution 4.0 International license.

10.1128/mSystems.00023-16.4 Figure S4  Vibrational characteristics of biomolecules in natural cheese in the pink area (red line) and outside the pink area (blue) line. Raman spectra were recorded at 532 nm. Download Figure S4, DOCX file, 0.1 MB. Copyright © 2016 Quigley et al. 2016 Quigley et al. This content is distributed under the terms of the Creative Commons Attribution 4.0 International license.

### Confirmation of the presence of *Thermus* in cheese.

As a consequence of the association between *Thermus* and samples of cheeses containing the pink discoloration defect, attempts were made to isolate this bacterium, which is not regarded as being a typical cheese-associated genus, from the defect-associated cheeses. Castenholz medium was employed, as it has previously been shown to support the growth of strains of *Thermus* (21), but due to its minimal nutrient content, it was unlikely to support the growth of other genera associated with cheese. Use of this approach resulted in the successful isolation of *Thermus* from a defect-associated cheese only but did not provide consistent counts. An alternate, culture-independent, qPCR-based method was developed to quantify *Thermus* organisms more accurately and more consistently. A primer pair for qPCR was designed with a view to selectively amplifying the polymerase I gene of *Thermus*. qPCR assays with a broad variety of controls established the primers to be specific. It was also speculated that the possible presence of residual *Thermus* DNA from *Taq* preparations might result in false positives, but this was found not to be the case. Quantitative PCR analysis (of the cheeses used for 16S rRNA gene analysis) confirmed that *Thermus* was absent from the control cheeses and that defect-associated cheeses contained on average 1.77 × 10^3^ CFU ⋅ g^−1^. Sequencing of PCR amplicons from defect-associated cheeses and from *Thermus* strains isolated from these cheeses revealed that the species in question was *T. thermophilus*. A representative defect-associated cheese isolate, *T. thermophilus* DPC6866, was employed in subsequent studies.

### *Thermus* is present at a number of locations in dairy processing plants.

To investigate how *T. thermophilus* becomes part of the cheese population, we undertook an environmental screen of two cheese processing facilities, during which swab and liquid samples were collected from around the plant (Table 1). The presence of *Thermus* in these samples was assessed using both culture-dependent and qPCR methods. While *Thermus* was detected in a number of samples, its presence was most consistently detected in hot water sources (Table 1).

**TABLE 1:** Environmental monitoring of *Thermus* organisms in dairy processing plants

**Area sample**(s)	**Sampling style**	Presence or absence of *Thermus* organisms as detected by:	
		**Culture**	**PCR**
Milk vat	Swab	–	–
Starter culture vats	Swab	–	–
Press vat	Swab	+	–
Starter cultures	Liquid	–	–
Hot water sample 1	Liquid	+	+
Hot water sample 2	Liquid	+	+
Hot water sample 3	Liquid	+	+
Brine before filtering	Liquid	–	–
Brine after filtering	Liquid	–	–
Antifungal dip	Liquid	–	+

### Addition of *T. thermophilus* DPC6866 recreates the pink discoloration defect in cheeses.

To establish definitively that *T. thermophilus* is responsible for the formation of pink defects in cheese, we produced cheese, at the pilot-scale level, according to the production protocol employed by the commercial producers of this Continental cheese type, to which *T. thermophilus* DPC6866 was added; then we compared the development of a pink discoloration with that of a control cheese. In each instance, four cheeses were produced, i.e., a control cheese which did not contain *T. thermophilus* and three experimental cheeses, all of which contained *T. thermophilus* at 10^6^ CFU ⋅ ml^−1^ but which contained different levels of starter bacteria. Experiment 1 (Exp1) contained starter cultures at standard inoculum levels, i.e., 0.055% *L. helveticus* DPC6865 (10^8^ CFU ⋅ ml^−1^), 0.11% *S. thermophilus* (10^8^ CFU ⋅ ml^−1^), and 0.00088% *P. freudenreichii* DPC6451 (10^8^ CFU ⋅ ml^−1^). The amounts of these cultures used in Exp2 and Exp3 cheeses were altered to reflect natural variations in starter levels. More specifically, Exp2 differed from Exp1 by virtue of higher-than-normal levels of *L. helveticus* inoculum (0.11%), while Exp3 also contained high levels of *L. helveticus* inoculum (0.11%) but had lower levels of *S. thermophilus* inoculum (0.055%) (Table 2). The numbers of the respective *S. thermophilus*, *L. helveticus*, PAB, and nonstarter lactic acid bacilli (NSLAB) in the cheese were monitored throughout the cheese production and ripening (116 days) process and were in line with expectations (Table S4; Fig. S5).

10.1128/mSystems.00023-16.5 Figure S5  (a) Counts of ripening bacteria, namely, *Lactobacillus helveticus* (Lh), *Streptococcus thermophilus* (St), propionic acid bacteria (PAB), and nonstarter lactic acid bacteria (NSLAB), throughout ripening, at 1 day (black bars with white dots), 11 days (stippled bars), 46 days (bars with hatch marks from top left to lower right), 60 days (bars with vertical lines), 88 days (bars with hatch marks from top right to lower left), and 116 days (bars with horizontal lines). (b) *Thermus thermophilus* levels, as determined by qPCR, throughout manufacture. M, inoculated milk; W, whey; C, curd. Bar results in each group appear in the following order (from left to right): Exp3 cheese, Exp1 cheese, and Exp2 cheese. (c) Effects of different treatments on cheese pH during ripening. Diamonds, control cheese; squares, experiment 1 cheese; triangles, experiment 2 cheese; crosses, experiment 3 cheese. (d) Effects of different experimental setups on the percentage of nitrogen soluble at pH 4.6 during the ripening time. Diamonds, control cheese; squares, experiment 1 cheese; triangles, experiment 2 cheese; crosses, experiment 3 cheese. (e) Effects of different experimental setups on free-amino-acid levels after 144 days of ripening. White bars, control cheese; gray bars, experiment 1 cheese; hatched bars, experiment 2 cheese; bars with horizontal lines, experiment 3 cheese. Download Figure S5, DOCX file, 0.2 MB. Copyright © 2016 Quigley et al. 2016 Quigley et al. This content is distributed under the terms of the Creative Commons Attribution 4.0 International license.

**TABLE 2:** Details and differences between procedures of manufacturing of Continental-type spiked-cheeses in the trials^a^

Cheese treatment	% of starter culture inoculum			Test bacterium *Thermus thermophilus* (CFU ⋅ ml^−1^)
	*Streptococcus* *thermophilus*	*Lactobacillus* *helveticus*	*Propionibacterium* *freudenreichii*	
Control	0.11	0.055	0.00088	0
Exp1	0.11	0.055	0.00088	10^6^
Exp2	0.11	0.11	0.00088	10^6^
Exp3	0.055	0.11	0.00088	10^6^

a The volume of milk was 454 kg. The curd formation was standard. The cooking temperature was increased 0.5°C per min until 45°C was reached and then it was increased 1°C per min until 53°C was reached. The drain pH was 6.30. Curds were placed in a prepress and a mold. A brine salting method was used. The cheese size was 10 kg. Cold room ripening was at 8.5°C for 10 days, warm room ripening was at 22°C for 7 weeks, and the cheese was stored at 4.5°C after the hot room step.

Visual examination of the cheeses revealed that the pinking defect was strongly evident in Exp2 cheese. The defect was quantified using a chroma meter to determine Hunter *a* values, which determine the level of redness (+) to greenness (−) (22). Through the center of the Exp2 cheese there was a shift toward a more positive average value (i.e., more red) that was not evident in the control cheese (Table 3). These differences were first noted after day 116 of ripening, and the relative difference in redness became more apparent by day 144. Indeed, the Hunter *a* values at day 144 for Exp2 were significantly less negative than those of the control cheese (*P* = 0.0009) cheese.

**TABLE 3:** Effect of treatment on color properties as determined by Hunter *L*, *a*, *b* dimensions^a^

Cheese sample	Area assessed	*a* value at 144 days
Control	Top	−2.22
	Side	−2.17
	Base	−2.32
	Center	−2.38
Exp1	Top	−2.21
	Side	−2.28
	Base	−2.21
	Center	−1.95
Exp2	Top	−2.18
	Side	−2.16
	Base	−2.10
	Center	−1.34^b^
Exp3	Top	−2.14
	Side	−2.35
	Base	−2.13
	Center	−1.82

a Data are means from three replicate trials. *a* values indicate the formation of redness. The results are those taken from 144-day-old cheeses.

b There was a statistically significant difference from the results for the control cheese (*P* = 0.0009).

### Starter, PAB, and NSLAB viability during cheese ripening.

Mean viable numbers of *S. thermophilus* cells were determined to be 10^7^ CFU ⋅ g^−1^ at day 1 of ripening in the control, Ex1, and Exp2 cheeses and 10^6^ CFU ⋅ g^−1^ in Exp3 cheese, which correlates with levels of the starter *S. thermophilus* inoculated into the cheese milk. There was a significant increase in numbers of *S. thermophilus* cells between 1 and 11 days of ripening (*P* = 0.0063); however, thereafter, there was no significant change (see Fig. S5 in the supplemental material), but there were no significant differences between treatments. *L. helveticus* numbers were 1 × 10^6^ CFU ⋅ g^−1^ at 1 day of ripening in control and Exp1 cheese, while numbers were 5 × 10^6^ CFU ⋅ g^−1^ in Exp2 and Exp3 cheeses, again reflecting the different levels of the *L. helveticus* starter added. The changes observed in levels of *L. helveticus* during cheese production were not significant. Counts of PAB increased significantly until 46 days of ripening (*P* < 0.0001) (Fig. S5); however, they did not differ significantly between treatments. Numbers of viable NSLAB increased significantly until the end of warm-room ripening (Fig. S5) (*P* < 0.0001). We observed a significant difference in the levels of NSLAB between control cheese and Exp2 cheese (*P* = 0.0438) and control cheese and Exp3 cheese (*P* = 0.0225) at 60 days of ripening. Using culture-independent qPCR, we determined the levels of *T. thermophilus* present in the inoculated milk, lost in whey, and retained in curd, as well as throughout ripening (Fig. S5). We established that *Thermus* was present at 10^6^ CFU ⋅ ml^−1^ in milk after a 1-h inoculation (sampled prior to rennet addition). There was some loss of *T. thermophilus* in whey, i.e., 10^2^ CFU ⋅ ml^−1^; however, considerable levels were retained within the curd (10^5^ CFU ⋅ g^−1^). Control cheeses, which were not spiked with *T. thermophilus*, were also assessed and were found not to contain *Thermus* organisms (data not shown), establishing that no natural contamination or cross-contamination occurred during production. Slight numerical increases in the levels of *T. thermophilus* cells were noted during hot-room ripening; however, these were not significant. Following transfer to the cold room for continued ripening, we observed a slight decrease in the levels of *T. thermophilus* to 10^4^ CFU ⋅ g^−1^. This was consistently observed for all three experimental cheeses (Fig. S5).

### Compositions of cheeses.

The gross compositions of cheeses at 11 days of ripening were assessed and are summarized in Table S1 in the supplemental material. All cheeses had statistically similar pH values, levels of moisture, salt distributions, and proteins. The consistency of these results between cheeses and cheese trials indicates good repetition across each day of manufacture; i.e., no significant differences were detected between these variables. Significant increases in pH, in the levels of nitrogen soluble at pH 4.6 (pH 4.6 SN), and in total free amino acids (FAAs) (*P* was <0.0001 for all three parameters assessed) were observed throughout ripening (Fig. S5). The concentrations of individual FAAs (in milligrams per kilogram of cheese) in all cheeses at 144 days of ripening are shown in Fig. S5. The FAAs present at the greatest concentrations in the cheeses at most ripening times were glutamic acid, valine, leucine, lysine, and proline and were in line with those expected in Continental-type cheeses (1).

## DISCUSSION

Metagenomic sequencing revealed a potential association between high levels of organisms of the genus *Thermus* and cheeses exhibiting a pink defect. *T. thermophilus* is a Gram-negative, extremely thermophilic, aerobic, nonpathogenic microorganism (23). It has been associated strongly with hot water sources, including springs (24) and tap water (25, 26), and was again detected in hot water supplies in this study. The identification of *Thermus* sp. as a major component of the pink-defect cheese microbiota highlights the merits of employing culture-independent strategies to investigate the biological basis for food defects. Representatives from this species can be difficult to culture and do not grow on the microbiological media routinely used to study or test cheese microbiota, thus explaining why this population has not previously been associated with the pinking phenomenon.

Bacteria from the phylum *Deinococcus*-*Thermus* are known for their resistance to extreme stresses, including radiation, oxidation, desiccation, and high temperature. When cultured, they typically have a red or yellow pigment because of their ability to synthesize carotenoids (23), which often act as nonenzymatic antioxidants and may thereby play a role as cellular protectants (23). Interestingly, members of this phylum, *Deinococcus* species and *Meiothermus* species, have been associated with pink-hue formation in various environments, including an undesirable discoloration of paper in paper manufacture industries (27, 28). Also, an ancient terrace, referred to as “The Pink Terraces,” which were recently rediscovered by geoscientists in New Zealand (Woods Hole Oceanographic Institution, Woods Hole, MA, USA), have a pink hue which has been attributed to the presence of the *Thermus ruber* bacterium (29). Analysis of shotgun metagenomic data revealed the presence of *Thermus* genes involved in carotenoid biosynthesis in defect-associated cheeses. More specifically, genes are involved in the formation of lycopene, a red pigment, and include *crtB* (phytoene synthase) and *crtI* (phytoene desaturase). Carotenoid production is a common feature of *Thermus* species, and there have been a number of studies in which carotenoid biosynthesis homologs in *Deinococcus*-*Thermus* species, including *T. thermophilus* HB8 and *T. thermophilus* HB27, have been characterized (23, 30, 31). Notably, these observations are consistent with our detection, through Raman analysis, of a carotenoid-associated peak within the pink region of defect-associated cheeses.

Following the detection of *Thermus* at high levels in cheeses with a pink defect, a series of cheese trials were carried out to determine whether the *T. thermophilus* bacterium is indeed responsible for this phenomenon. For this, we inoculated cheese with *T. thermophilus* and with thermophilic starter bacteria at various levels. The levels of *T. thermophilus* introduced were consistent with that of a previous study which established that the inoculation of a milk supply with *T. thermophilus* N8, itself a dairy isolate, in the range of 5 to 100 CFU/ml milk prior to its being passaged through a tube heat exchanger resulted in the strain both adhering to and growing within the tube heat exchanger to levels in excess of 1.2 × 10^7^ CFU/cm^2^ even at a high temperature (83°C). This study also describes heat exchangers as potential reservoirs for milk contamination (32).

Following production of the cheeses, no differences were noted in the chemical compositions of the various cheeses. This is consistent with the results of previous studies which also failed to find a correlation between cheese compositional profiles, including profiles relating to moisture, salt, soluble nitrogen, and free amino acids, and the development of the pink defect (5, 12, 33). Through an assessment based on colorimetric analysis and from visual examination, greater levels of “pinking” were apparent in the cheeses in which *T. thermophilus* is present than in cheeses in which the organism was not present. Notably, in situations where the levels of starter cultures were adjusted, particularly where the *L. helveticus* inoculum was increased but the *S. thermophilus* inoculum was unaltered, the pink color formation was more intense.

The biological basis for the contribution of increased proportions of lactobacilli to the pinking phenomenon has yet to be determined but may be that other components of the cheese microbiota influence carotenoid production or modification to intensify the associated pink discoloration. This will be addressed in future studies. Regardless, these findings have the potential to lead to the development of strategies to understand the exact mechanism involved in *Thermus*-mediated pink-defect formation in cheese, with the eventual goal being to eliminate the problem of pink discoloration in cheese and the associated economic loss.

## MATERIALS AND METHODS

### DNA extraction from cheeses.

Cheese samples (*n =* 18) from the same lot, with (9 defect-associated cheeses) or without (9 control cheeses) pinking discoloration, were sourced. For nucleic acid extraction, 1 g of cheese from the defect-associated or control cheese was combined with 9 ml of 2% trisodium citrate and homogenized before DNA was extracted using the PowerFood microbial DNA isolation kit (Mo Bio Laboratories Inc., CA, USA) (34) as described previously (34). Additional steps were added to the standard manufacturer’s instructions. These included treatment of the homogenate with 50 µg ⋅ ml^−1^ lysozyme (Sigma-Aldrich Ltd., Arklow, Wicklow, Ireland) and 100 U mutanolysin (Sigma Ltd.) at 37°C for 1 h, followed by protein digestion by addition of 250 µg ⋅ ml^−1^ proteinase K (Sigma Ltd.) and incubation at 55°C for 1 h.

### Generation of 16S rRNA gene amplicons for high-throughput sequencing.

DNA extracts were used as a template for PCR amplification of 16S rRNA genes tags (V4-V5 region; 408 nucleotides long) using universal 16S rRNA primers predicted to bind to 94.6% of all 16S rRNA genes, i.e., the forward primer F1, 5′-AYTGGGYDTAAAGNG-3′ (RDP’s Pyrosequencing Pipeline [http://pyro.cme.msu.edu/pyro/help.jsp]), and the reverse primer V5, 5′-CCGTCAATTYYTTTRAGTTT-3′ (35). The primers incorporated the proprietary 19-mer sequences at the 5′ end to allow emulsion-based clonal amplification for the 454 pyrosequencing system. Unique molecular identifier (MID) tags were incorporated between the adaptamer and the target-specific primer sequence to allow identification of individual sequences from pooled amplicons. The PCR mixture contained 25 µl BioMix Red (Bioline Reagents Ltd., London, United Kingdom), 1 µl each primer (10 pmol), 5 µl DNA template, and enough nuclease-free H_2_O to give a final reaction volume of 50 µl. PCR amplification was performed using a G-Storm thermal cycler (Somerset Biotechnology Centre, Somerset, United Kingdom). The amplification program consisted of an initial denaturation step at 94°C for 2 min, followed by 40 cycles of denaturation at 94°C for 1 min, annealing at 52°C for 1 min, and an extension at 72°C for 1 min. A final elongation step at 72°C for 2 min was also included. Amplicons were cleaned using the AMPure XP purification system (Beckman Coulter, Takeley, United Kingdom). The quantity of DNA was assessed using the Quant-It PicoGreen double-stranded-DNA (dsDNA) reagent (Invitrogen, Carlsbad, CA) in accordance with the manufacturer’s instructions and a NanoDrop 3300 fluorospectrometer (Thermo Fisher Scientific Inc., Waltham, MA). The NanoDrop 3300 fluorospectrometer excites in the presence of dsDNA bound with PicoGreen at 470 nm and monitors emission at 525 nm.

### 16S rRNA gene sequencing and bioinformatic analysis.

The 16S rRNA gene V4-V5 amplicons were sequenced on a 454 genome sequencer FLX platform (Roche Diagnostics Ltd., Burgess Hill, West Sussex, United Kingdom) according to 454 pyrosequencing protocols. Read processing was performed using techniques implemented in the RDP Pyrosequencing Pipeline (36). Sequences not passing the FLX quality controls were discarded, the 454 pyrosequencing-specific portions of the primer were trimmed, and the raw sequences were sorted according to tag sequences; reads with low quality scores (quality scores below 40) and short lengths (less than 150 bp for the 16S rRNA gene V4 region) were removed, as were reads that did not have exact matches with the primer sequence. The QIIME suite of programs was used to align, chimera check, cluster, and measure microbial α-diversities and to plot rarefaction curves to determine if sequencing was carried out to sufficient depths (37). Taxonomy was assigned to trimmed FASTA sequences using BLAST (38) against the SILVA version 100 database (39). The resulting BLAST output was parsed using MEGAN version 4.3.0 (40). MEGAN assigns reads to NCBI taxonomies by employing the lowest-common-ancestor algorithm, which assigns each RNA tag to the lowest common ancestor in the taxonomy from a subset of the best-scoring matches in the BLAST result. Bit scores were used from within MEGAN for filtering the results (BLAST bit-score, 86) (41)

The statistical significance of differences in proportions of microbial taxa was determined by the nonparametric Kruskal-Wallis test (42) using the Minitab statistical package; the level of significance was determined at a *P* of <0.05. 

### Shotgun metagenomic sequencing and gene function analysis.

Additional defect-associated and control cheeses were shotgun sequenced for metagenomic analysis. This work was carried out by GATC Biotech (Constance, Germany), as was DNA extraction from cheese samples and DNA library preparations, which were sequenced on an Illumina HiSeq 2000 platform (single 150-bp reads). Resultant reads were processed using Picard/SAM tools and assembled using Velvet. Genes were then predicted using MetaGeneMark and annotated using the BLAST program against the NR database. Finally, sequences were parsed using MEGAN version 5.7.1 (40), and gene function was assessed using the KEGG database (43).

### Raman analysis.

Raman spectra were acquired with a Raman integrated scanning electron (RISE) microscope integrating the TESCAN dual-beam (FIB-SEM) GAIA system with a WITec confocal Raman microscope. The 532-nm green laser was used for spectral acquisition. The integration time per pixel was 0.5 s. An area of interest was imaged with 3 steps per 1 µm (step size, 1/3 µm). Spectra were processed by ProjectPlus software (WITec). First the principal-component analysis (PCA) procedure was run to find the number of components, and then nonnegative matrix factorization (NMF) was applied to distinguish the spectra of the components.

### Culturing of *Thermus.*

Castenholz tryptone yeast extract (TYE) medium was chosen to selectively support the growth of strains from the genus *Thermus*. Castenholz TYE medium was prepared by mixing 5 parts 2× Castenholz salts with one part 1% TYE and 4 parts distilled water. An enrichment step, during which cheese was homogenized in Castenholz medium and incubated at 70°C for 3 days, was employed to encourage the growth of *Thermus* organisms, which are characterized by their highly thermophilic nature, and to prevent the growth of more moderately thermophilic cultures, such as those within the starter culture population. A 3% agar was employed to allow incubation at high temperature (55°C) without rapid dehydration of the media. Castenholz Salts (2×) contained 0.2 g nitrilotriacetic acid, 0.12 g CaSO_4_⋅2H_2_O, 0.2 g MgSO_4_⋅H_2_O, 0.016 g NaCl, 0.21 g KNO_3_, 1.4 g NaNO_3_, 0.22 g Na_2_HPO_4_, 2.0 ml FeCl_3_ solution (0.03%), and 2.0 ml Nitsch’s trace elements (0.5 ml H_2_SO_4_, 2.2 g MnSO_4_, 0.5 g ZnSO_4_⋅7H_2_O, 0.5 g H_3_BO_3_, 0.016 g CuSO_4_⋅5H_2_O, 0.025 g Na_2_MoO_4_⋅2H_2_O, 0.046 g CoCl_2_⋅6H_2_O distilled water [1 liter]), adjusted to a final volume of 1 liter and a final pH of 8.2. One percent TYE solution consisted of 10.0 g tryptone, 10.0 g yeast extract dissolved in 1 liter distilled water. The final pH of Castenholz TYE medium was 7.6. For preparation of the corresponding agar, 3% (wt/vol) bacteriological agar was added to the final solution.

### PCR- and qPCR-based detection of *Thermus.*

A set of primers (TpolFor, 5′-AGCCTCCTCCACGAGTTC-3′, and TpolRev, 5′-GTAGGCGAGGAGCATGGGGT-3′) targeting a region specifically conserved within the polymerase I gene of *Thermus* were designed to facilitate PCR and qPCR-based detection of the genus. The theoretical specificities of these primers were tested using the oligonucleotide probe search tools in the BLAST classifier database (38). The PCR mixture contained 25 µl BioMix Red (Bioline Reagents Ltd., London, United Kingdom), 1 µl each primer (10 pmol), 5 µl DNA template, and enough nuclease-free H_2_O to give a final reaction volume of 50 µl. PCR amplification of the polymerase I gene using these primers was carried out under the following parameters: 95°C for the initial 2-min denaturation, followed by 40 cycles of 94°C for 30 s, 63°C for 30 s, and 72°C for 45 s, with a final elongation of 72°C for 2 min. The resultant products were visualized by agar gel electrophoresis. Amplicons generated were cleaned using the Roche High Pure PCR cleanup kit and sequenced (Source Bioscience, Dublin, Ireland). The specificity of the primer pair was tested using DNA from a selection of cheese-associated Gram-positive and Gram-negative cultures, i.e., *Streptococcus thermophilus*, *Lactobacillus helveticus* DPC6865, *Propionibacterium freudenreichii* DPC6451, and *Lactococcus lactis* HP, as well as *Escherichia coli* DPC6009, *Listeria monocytogenes* EGD-e, *Salmonella enterica* serovar Typhimurium LT2, and *Bifidobacterium longum* DPC5697 (all strains were obtained from the Moorepark Culture Collection, Fermoy, Cork, Ireland).

To facilitate the quantification of *Thermus* by molecular means, a qPCR protocol was designed. Genomic DNA was extracted from *Thermus thermophilus* HB27 (DSMZ Culture Collection, Germany) using the PowerFood microbial DNA extraction kit (Mo Bio Laboratories Inc.). A PCR product from within the polymerase I gene was generated using the genus-specific primers, as described above.

Purified amplicons were cloned into the pCR2.1-TOPO vector using the TOPO-TA cloning system (Invitrogen, Life Technologies, Carlsbad, CA) in accordance with the manufacturer’s instructions. Following cloning, the complete construct was transformed into chemically competent TOP-10 *E. coli* cells (Invitrogen) and harvested on LB media containing 100 µg ⋅ ml^−1^ ampicillin. The accuracy of the cloned amplicon was confirmed by restriction analysis and DNA sequencing. Quantitative PCR standards were prepared after the linearization of plasmid DNA with the PstI restriction enzyme and quantification with the NanoDrop ND-1000 (Thermo Fisher Scientific Inc.). A standard curve was then generated via a series of dilutions from 10^2^ to 10^8^ copies µl^−1^ DNA. The LightCycler 480 SYBR green I master kit (Roche Diagnostics Ltd.) was used for quantification according to the manufacturer’s instructions. Each PCR mixture contained 5 µl Sybr green master mix (Roche Diagnostics Ltd.), 1 µl both the forward and the reverse primer (7.5 pmol), and 2 µl DNA, and each mixture was made up to a final volume of 10 µl with nuclease-free single-distillation H_2_O. The PCR conditions were as follows: an initial denaturation at 95°C for 10 min, followed by 45 cycles of denaturation at 95°C for 20 s, annealing at 61°C for 15 s, and elongation 72°C for 20 s. Assays were performed in triplicate. To facilitate quantification by qPCR, we applied the formula of Quigley et al. (44) to convert from the number of copies per microliter to the number of CFU per gram of cheese.

### Environmental sampling for the presence of *Thermus* organisms in dairy processing plants.

Monitoring for the presence of *Thermus* was carried out at two cheese manufacturing facilities; we assessed swabs of various surfaces and liquid samples from various sources throughout the facilities. Samples that were collected included swabs of surface areas covering starter culture vats, milk vats, mixing vats, pressing vats, and water hoses. Liquid samples included water sources and pre- and postbrining solutions. Also assessed were antifungal dips and batch starter cultures. For all samples, culture-based and culture-independent *Thermus* detection methods were applied as described above.

### Cheese manufacture and analysis.

Cheese manufacture incorporated three replicate trials consisting of four treatments (a control and three test treatments), each of which required 454 kg of milk (i.e., a combined total of 5,448 kg of milk). Three 10-kg rounds of cheese were produced per treatment. The scale and conditions used in this study were reflective of those used during commercial cheese manufacture. The starter cultures *S. thermophilus* (defined starter mix; Laboratories Standa, Caen, France) and *L. helveticus* DPC6865 (Moorepark Culture Collection) were each grown overnight at 37°C in reconstituted low-heat skim milk powder, which had first been heat treated at 90°C for 30 min. *Propionibacterium freudenreichii* DPC6451 (Moorepark Culture Collection) was grown for 3 days at 30°C in sodium lactate broth. *T. thermophilus* DPC6866 (Moorepark Culture Collection), obtained from a cheese with a pink defect, was grown in Castenholz broth at 60°C with shaking for 36 h. Cells were collected by centrifugation at 14,000 × *g* for 20 min, washed once to remove trace media, and resuspended in sterile water. Raw milk was obtained from a Teagasc, Moorepark, dairy herd, standardized, pasteurized at 72°C for 15 s, and pumped at 32°C into four individual cylindrical stainless steel vats with automated variable-speed cutters and stirrers. This milk was employed to manufacture a Continental-type cheese at a pilot-scale level at Moorepark Technology Ltd. (Fermoy, Cork, Ireland). To enumerate specific bacterial components, cheese samples were aseptically removed, placed in a stomacher bag, diluted 1:10 with sterile trisodium citrate (2%, wt/vol; Sigma Ltd., Arklow, Wicklow, Ireland), and homogenized in a Seward Stomacher 400 lab system (Seward Ltd., West Sussex, United Kingdom) for 2 min. Further dilutions were prepared as required. Viable *S. thermophilus* cells were enumerated on M17 agar (Oxoid Ltd., Hampshire, United Kingdom) with 0.5% lactose (Oxoid Ltd.) at 42°C for 3 days. *L. helveticus* cells were enumerated on MRS agar (Oxoid Ltd.) adjusted to pH 5.4, at 37°C for 3 days under anaerobic conditions. PAB levels were enumerated on sodium lactate agar containing 40 µg ⋅ ml^−1^ kanamycin (Sigma Ltd.) at 30°C for 7 days under anaerobic conditions. Nonstarter lactic acid bacteria (NSLAB) were enumerated on *Lactobacillus* selective agar (LBS agar; Difco) at 30°C for 5 days aerobically. Details with respect to the manufacture of control and test cheeses can be found in Table 2. Microbiological cells were enumerated, compositions of cheeses were determined, and proteolysis was measured at various stages of ripening (Tables S4 and S5). *T. thermophilus* was monitored using qPCR methods. To facilitate this, DNA was extracted from milk, whey, or 10 ml cheese homogenate using the PowerFood DNA isolation kit as described above. Grated samples from cheeses were analyzed for salt (45), moisture (46), and protein (47) after 11 days of manufacture; pH (48) was measured throughout ripening. The levels of nitrogen soluble at pH 4.6 (pH 4.6 SN) were measured as described by Sheehan et al. (49). Free-amino-acid analysis was carried out on pH 4.6 SN extract as described by Fenelon et al. (50).

### Visual detection of pinking.

Cheese wheels were examined visually throughout ripening for the formation of the pink discoloration defect. Pink-defect formation was quantified using a colorimeter (CR-400 chroma meter; Konica Minolta, Osakam, Japan) using the Hunter *L*, *a*, *b* color scale. The color was measured on the surfaces of freshly sliced exposed cheese. The colorimeter was standardized using the white Konica Minolta calibration plate for the following color space parameters: Y, y, and x, as defined by the International Commission on Illumination. Hunter *a* (redness) values were recorded.

### Statistical analysis.

A randomized complete block design that incorporated the four treatments and three block treatments (replicate trials) was used for the analysis of response variables relating to the compositions of cheeses and their moisture, salt, and protein, as well as for the analysis of starter bacteria, PAB, NSLAB, *T. thermophilus*, pH, pH 4.6 SN, free amino acids (FAA), and apparent color differences. Analysis of variance was carried out on data using the general linear model procedure of SAS (SAS Institute, Cary, NC). The Tukey honestly significant difference test was used to determine the significance of differences between the means. The level of significance was determined at a *P* of <0.05.

### Accession number(s).

Sequence data have been deposited in the European Nucleotide Archive (ENA) under accession number PRJEB6952.
