# Supplementary material for: Thermus and the Pink Discoloration Defect in Cheese
Source: mSystems. 2016 Jun 14;1(3):e00023-16. doi: 10.1128/mSystems.00023-16 (PMC5069761; doi:10.1128/mSystems.00023-16)
Supplement: Figure S1 [file sys003162029sf1.docx]

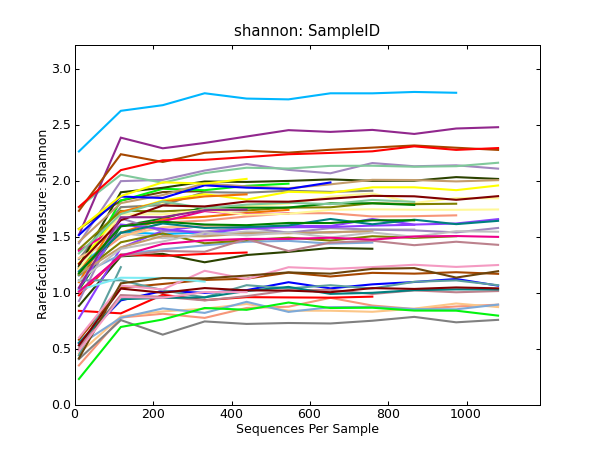
**Figure S1:** **16S rRNA sequencing reads analysis**

16S reads per cheese ≥3,500 (average number of reads per sample was 3960). Rarefaction curve of α-diversity, represented by Shannon indices, for all samples sequenced confirmed that satisfactory coverage was achieved (Figure S1). Sequence data has been uploaded to European Nucleotide Archive (ENA) accession number PRSEB6952.
