# Supplementary material for: Thermus and the Pink Discoloration Defect in Cheese
Source: mSystems. 2016 Jun 14;1(3):e00023-16. doi: 10.1128/mSystems.00023-16 (PMC5069761; doi:10.1128/mSystems.00023-16)
Supplement: Table S1 [file sys003162029st6.docx]

**Table S1:** Average % 16S rRNA reads assigned at genus level to control and defect cheeses

| **Genus** | **Control** | **Defect** |
| --- | --- | --- |
| *Anoxybacillus* | 0.00 | 0.08 |
| *Streptococcus* | 37.15 | 26.98 |
| *Lactococcus* | 0.52 | 0.60 |
| *Enterococcus* | 0.04 | 0.01 |
| *Lactobacillus* | 61.70 | 65.52 |
| *Clostridium* | 0.04 | 0.00 |
| *Catenibacterium* | 0.00 | 0.29 |
| *Carnobacterium* | 0.04 | 0.01 |
| *Thermus* | 0.05 | 6.44 |
| *Propionibacterium* | 0.48 | 0.07 |
