# Supplementary material for: Thermus and the Pink Discoloration Defect in Cheese
Source: mSystems. 2016 Jun 14;1(3):e00023-16. doi: 10.1128/mSystems.00023-16 (PMC5069761; doi:10.1128/mSystems.00023-16)
Supplement: Figure S2 [file sys003162029sf2.docx]

**Figure S2: Bacterial composition of defect and control cheese as determined by shotgun metagenomic sequencing** Sequences assigned according to MEGAN at the Phylum level for cheese affected by the pink discolouration defect and corresponding control cheeses.
