# Supplementary material for: Thermus and the Pink Discoloration Defect in Cheese
Source: mSystems. 2016 Jun 14;1(3):e00023-16. doi: 10.1128/mSystems.00023-16 (PMC5069761; doi:10.1128/mSystems.00023-16)
Supplement: Table S2 [file sys003162029st7.docx]

**Table S2:** BLAST of degenerate primers used in 454 compositional sequencing against *P. freudenreichii* subsp. *shermanii* genome. The absence of similarity across the Forward primer explains the differences between *Propionibacteria* populations detected *via* compositional and shotgun sequencing.

| **Primer** | **Sequence** | **BLAST Template** | **Max Score** | | **Total Score** | **Query Cover** | **E-value** | **Identity** | **Accession** |
| --- | --- | --- | --- | --- | --- | --- | --- | --- | --- |
| Forward Primer | AYTGGGYDTAAAGNG | *P. freudenreichii* | | No Similarity | | | | | |
| V5-Reverse | CCGTCAATTYYTTTRAGTTT | *P. freudenreichii* | | 31.2 | 47.1 | 100% | 0.01 | 85% | LN624749.1 |
