# Supplementary material for: Thermus and the Pink Discoloration Defect in Cheese
Source: mSystems. 2016 Jun 14;1(3):e00023-16. doi: 10.1128/mSystems.00023-16 (PMC5069761; doi:10.1128/mSystems.00023-16)
Supplement: Table S3 [file sys003162029st8.docx]

**Table S3a:** Assembled shotgun metagenomic sequences per sample assigned at genus level to control and defect cheeses (as a % of those assigned)

| **Genus** | **Control 1** | **Control 2** | **Defect 1** | **Defect 2** | **Defect 3** | **Defect 4** | **Defect 5** | **Defect 6** | **Defect 7** | **Defect 8** |
| --- | --- | --- | --- | --- | --- | --- | --- | --- | --- | --- |
| *Lactobacillus* | 51.50 | 30.89 | 37.86 | 50.52 | 48.42 | 38.06 | 37.36 | 22.40 | 22.34 | 36.21 |
| *Lactococcus* | 0.11 | 0.00 | 0.00 | 0.00 | 0.00 | 0.00 | 0.00 | 0.00 | 0.00 | 0.00 |
| *Streptococcus* | 48.39 | 34.31 | 16.17 | 21.61 | 22.78 | 17.60 | 17.72 | 24.95 | 24.70 | 19.64 |
| *Propionibacterium* | 0.00 | 34.80 | 19.28 | 0.35 | 0.44 | 20.37 | 20.54 | 23.43 | 23.67 | 18.23 |
| *Deinococcus* | 0.00 | 0.00 | 0.00 | 0.00 | 0.00 | 0.00 | 0.00 | 0.00 | 0.00 | 0.18 |
| *Meiothermus* | 0.00 | 0.00 | 0.10 | 0.00 | 0.00 | 0.00 | 0.00 | 0.11 | 0.00 | 0.00 |
| *Thermus* | 0.00 | 0.00 | 26.17 | 27.40 | 28.24 | 23.87 | 24.29 | 29.10 | 29.28 | 25.74 |
| *Anoxybacillus* | 0.00 | 0.00 | 0.30 | 0.00 | 0.00 | 0.00 | 0.00 | 0.00 | 0.00 | 0.00 |
| *Clostridium* | 0.00 | 0.00 | 0.12 | 0.12 | 0.11 | 0.10 | 0.10 | 0.00 | 0.00 | 0.00 |

**Table S3b:** Assembled shotgun metagenomic sequences assigned per sample at species level to control and defect cheese (as a % of those assigned)

| **Species** | **Control**  **1** | **Control**  **2** | **Defect**  **1** | **Defect**  **2** | **Defect**  **3** | **Defect**  **4** | **Defect**  **5** | **Defect**  **6** | **Defect**  **7** | **Defect**  **8** |
| --- | --- | --- | --- | --- | --- | --- | --- | --- | --- | --- |
| *Lb. helveticus* | 75.91 | 28.14 | 16.73 | 26.12 | 27.93 | 17.64 | 17.70 | 23.34 | 23.56 | 16.29 |
| *Lb. iners* | 0.32 | 0.00 | 0.00 | 0.00 | 0.00 | 0.00 | 0.00 | 0.00 | 0.00 | 0.00 |
| *Lb. delbrueckii* | 0.00 | 0.00 | 29.39 | 47.22 | 43.81 | 27.77 | 27.11 | 0.80 | 0.39 | 29.15 |
| *Lb. casei* | 0.00 | 0.21 | 0.00 | 0.30 | 0.00 | 0.00 | 0.00 | 0.00 | 0.00 | 0.00 |
| *Lb. rhamnosus* | 0.24 | 0.00 | 0.00 | 0.00 | 0.00 | 0.00 | 0.00 | 0.00 | 0.00 | 0.00 |
| *L. lactis* | 0.24 | 0.00 | 0.00 | 0.00 | 0.00 | 0.00 | 0.00 | 0.00 | 0.00 | 0.00 |
| *S. agalactiae* | 0.00 | 0.18 | 0.00 | 0.00 | 0.00 | 0.00 | 0.00 | 0.00 | 0.00 | 0.00 |
| *S. caballi* | 0.40 | 0.00 | 0.00 | 0.00 | 0.00 | 0.00 | 0.00 | 0.00 | 0.00 | 0.00 |
| *S. infantarius* | 0.48 | 0.23 | 0.00 | 0.00 | 0.00 | 0.00 | 0.00 | 0.00 | 0.00 | 0.00 |
| *S. salivarius* | 1.03 | 0.29 | 0.00 | 0.00 | 0.00 | 0.00 | 0.00 | 0.39 | 0.39 | 0.27 |
| *S. thermophilus* | 21.39 | 8.22 | 4.37 | 6.95 | 7.69 | 4.68 | 4.73 | 7.54 | 7.55 | 5.11 |
| *P. acidipropionici* | 0.00 | 0.32 | 0.19 | 0.00 | 0.00 | 0.20 | 0.20 | 0.27 | 0.27 | 0.00 |
| *P. freudenreichii* | 0.00 | 62.18 | 34.74 | 0.49 | 0.70 | 36.50 | 36.87 | 48.66 | 49.04 | 33.91 |
| *P. acidifaciens* | 0.00 | 0.23 | 0.00 | 0.00 | 0.00 | 0.00 | 0.00 | 0.00 | 0.00 | 0.00 |
| *D. geothermalis* | 0.00 | 0.00 | 0.00 | 0.00 | 0.00 | 0.00 | 0.00 | 0.00 | 0.00 | 0.29 |
| *T.aquaticus* | 0.00 | 0.00 | 0.33 | 0.33 | 0.32 | 0.22 | 0.24 | 0.32 | 0.30 | 0.42 |
| *T. oshimai* | 0.00 | 0.00 | 0.21 | 0.22 | 0.26 | 0.00 | 0.00 | 0.34 | 0.27 | 0.00 |
| *T. scotoductus* | 0.00 | 0.00 | 0.87 | 1.14 | 0.97 | 0.73 | 0.70 | 1.05 | 1.03 | 0.95 |
| *T. sp. RL* | 0.00 | 0.00 | 0.94 | 1.30 | 1.26 | 0.80 | 0.74 | 1.37 | 1.25 | 1.09 |
| *T. sp. WG* | 0.00 | 0.00 | 0.00 | 0.00 | 0.23 | 0.00 | 0.00 | 0.00 | 0.00 | 0.00 |
| *T. thermophilus* | 0.00 | 0.00 | 12.00 | 15.94 | 16.82 | 11.45 | 11.70 | 15.93 | 15.94 | 12.50 |
| *Anoxybacillus* sp. SK3-4 | 0.00 | 0.00 | 0.24 | 0.00 | 0.00 | 0.00 | 0.00 | 0.00 | 0.00 | 0.00 |
|  |  |  |  |  |  |  |  |  |  |  |
