# Supplementary material for: Thermus and the Pink Discoloration Defect in Cheese
Source: mSystems. 2016 Jun 14;1(3):e00023-16. doi: 10.1128/mSystems.00023-16 (PMC5069761; doi:10.1128/mSystems.00023-16)
Supplement: Table S4 [file sys003162029st9.docx]

Table S4: Assessment carried out at different stages of manufacture and ripening.

| **Ripening Time (days)** | **Stages of Ripening** | **Sample Type** | **Microbiological Analysis** | **Compositional Analysis** |
| --- | --- | --- | --- | --- |
| 0 | Day of manufacture | Milk, Wey, Curd | Tt | pH |
| 1 | After Brining | Cheese | Tt, St, Lh, PAB | pH, Moisture, Salt, Proteins, pH4.6SN, FAA |
| 11 | After 10 days at cool room ripening (8.5°C) | Cheese | Tt, St, Lh, PAB, NSLAB | pH, Moisture, Salt, Proteins, pH4.6SN, FAA |
| 46 | After 5 weeks at warm room ripening (22°C) | Cheese | Tt, St, Lh, PAB, NSLAB | pH, pH4.6SN, FAA, visual examination |
| 60 | End of warm room ripening (22°C) | Cheese | Tt, PAB, NSLAB | pH, pH4.6SN, FAA, visual examination |
| 88 | After 1 month in cold room (4.5°C) | Cheese | Tt, NSLAB | pH, pH4.6SN, FAA, visual examination |
| 116 | After 2 months in cold room (4.5°C) | Cheese | Tt, NSLAB | pH, pH4.6SN, FAA, visual examination |
| 144 | After 3 months in cold room (4.5°C) | Cheese | Tt | pH, pH4.6SN, FAA, visual examination |

Tt – *Thermus thermophilus*; St – *Streptococcus thermophilus*; Lh – *Lactobacillus helveticus*; PAB – Propionic Acid Bacteria; NSLAB – Non-starter lactic acid bacteria; pH4.6SN – pH4.6 soluble nitrogen FAA – Free Amino Acid
