# Supplementary material for: Thermus and the Pink Discoloration Defect in Cheese
Source: mSystems. 2016 Jun 14;1(3):e00023-16. doi: 10.1128/mSystems.00023-16 (PMC5069761; doi:10.1128/mSystems.00023-16)
Supplement: Table S5 [file sys003162029st10.docx]

Table S5: Composition of cheeses at 11 days post manufacture.

|  | **pH** | **% Moisture** | **% Salt** | **% Protein** |
| --- | --- | --- | --- | --- |
| **Control** | 5.21 | 41.10 | 1.36 | 24.931 |
| **Exp 1** | 5.24 | 40.80 | 1.25 | 25.271 |
| **Exp 2** | 5.21 | 41.50 | 1.22 | 25.723 |
| **Exp 3** | 5.23 | 40.94 | 1.28 | 24.804 |

Data presented in this table are means for three replicate trials.
