# Supplementary material for: Thermus and the Pink Discoloration Defect in Cheese
Source: mSystems. 2016 Jun 14;1(3):e00023-16. doi: 10.1128/mSystems.00023-16 (PMC5069761; doi:10.1128/mSystems.00023-16)
Supplement: Figure S3 [file sys003162029sf3.docx]

**Figure S3: Breakdown of KEGG pathways present**. Bar graph data is represented in percentage of assigned read
