# Supplementary material for: Thermus and the Pink Discoloration Defect in Cheese
Source: mSystems. 2016 Jun 14;1(3):e00023-16. doi: 10.1128/mSystems.00023-16 (PMC5069761; doi:10.1128/mSystems.00023-16)
Supplement: Figure S4 [file sys003162029sf4.docx]

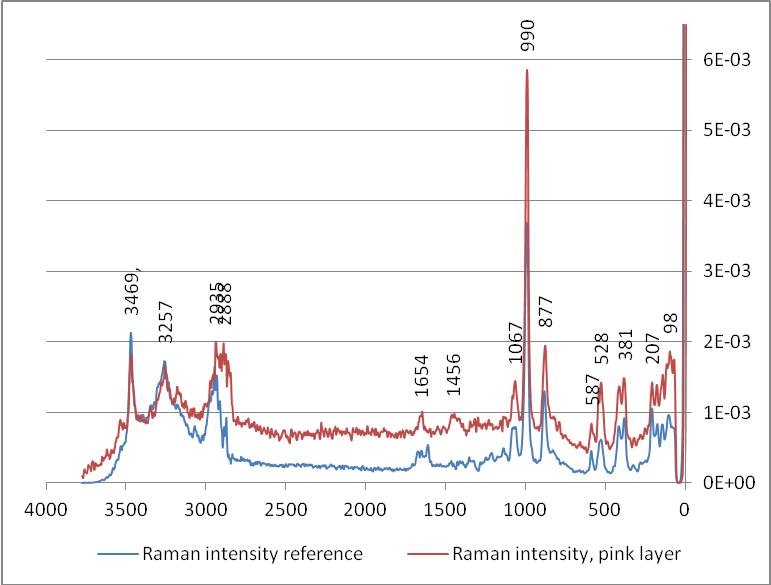


**Figure S4:** Vibrational characteristics of biomolecules in natural cheese in the pink area (red line) and outside the pink area (blue) line, Raman spectra recorded at 532 nm.
