# Supplementary material for: Thermus and the Pink Discoloration Defect in Cheese
Source: mSystems. 2016 Jun 14;1(3):e00023-16. doi: 10.1128/mSystems.00023-16 (PMC5069761; doi:10.1128/mSystems.00023-16)
Supplement: Figure S5 [file sys003162029sf5.docx]

Figure S5a: Counts of ripening bacteria, *Lactobacillus helveticus* (Lh), *Streptococcus thermophilus* (St), propionic acid bacteria (PAB) and non-starter lactic acid bacteria (NSLAB) throughout ripening, 1d , 11d , 46 d , 60 d , 88 d , 116 d .

**Figure S5b:**  *Thermus thermophilus* levels, as determined by qPCR, throughout manufacture. M-inoculated milk, W-whey, C-curd. Experimental cheese 1 , experimental cheese 2 , experimental cheese 3 .


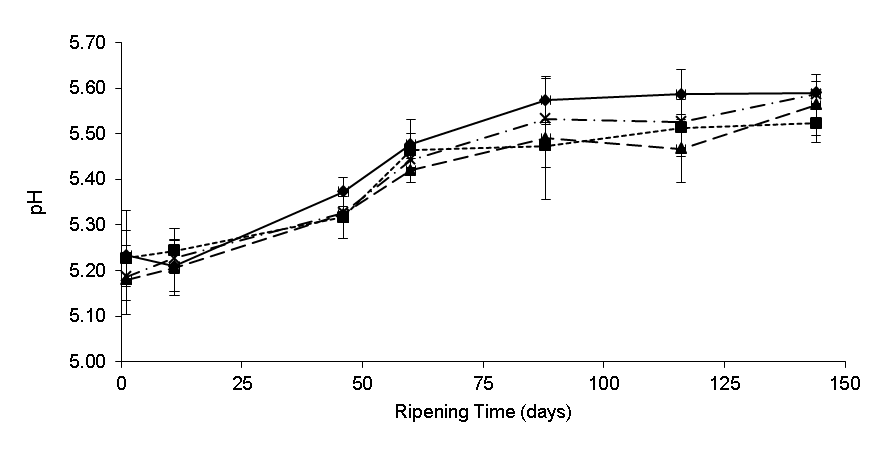


**Figure S5c:** The effect of different treatments on cheese pH over ripening. Control cheese
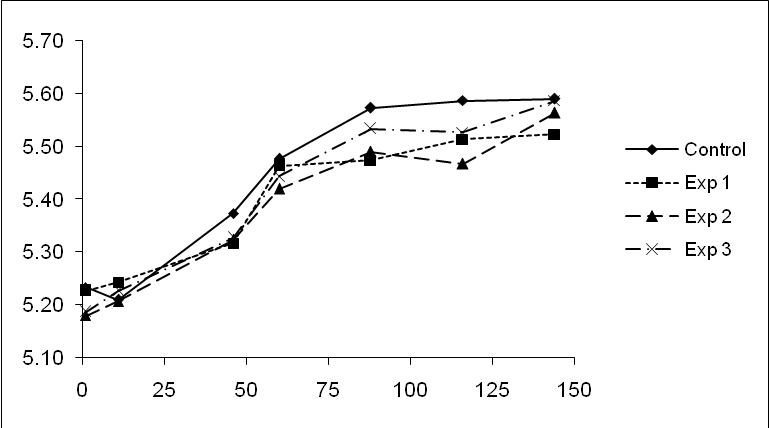
, experiment 1 cheese
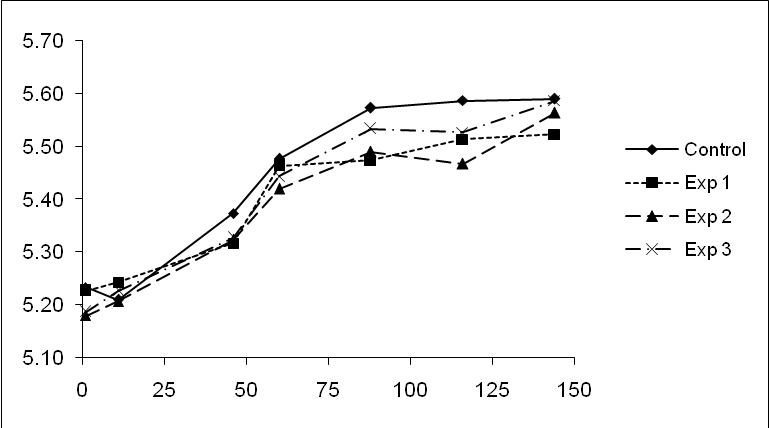
, experiment 2 cheese
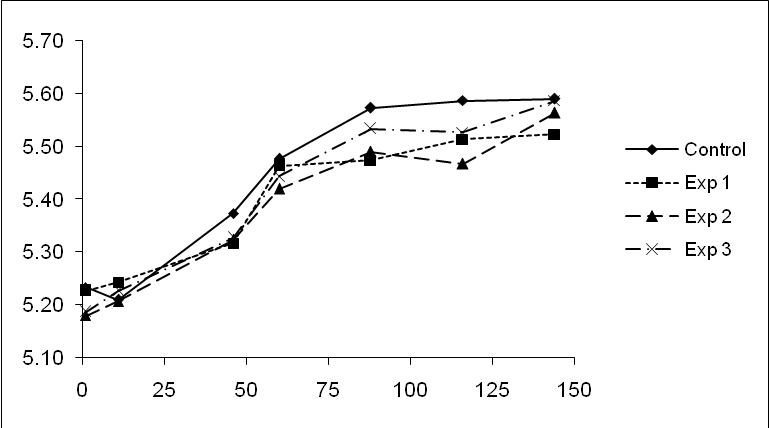
 and experiment 3 cheese
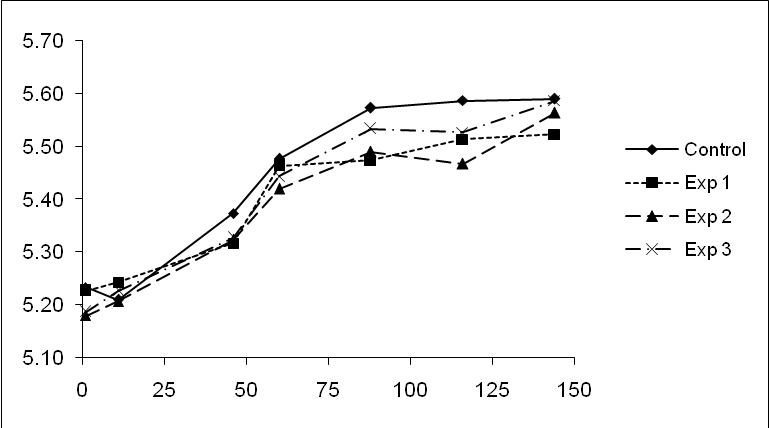
.


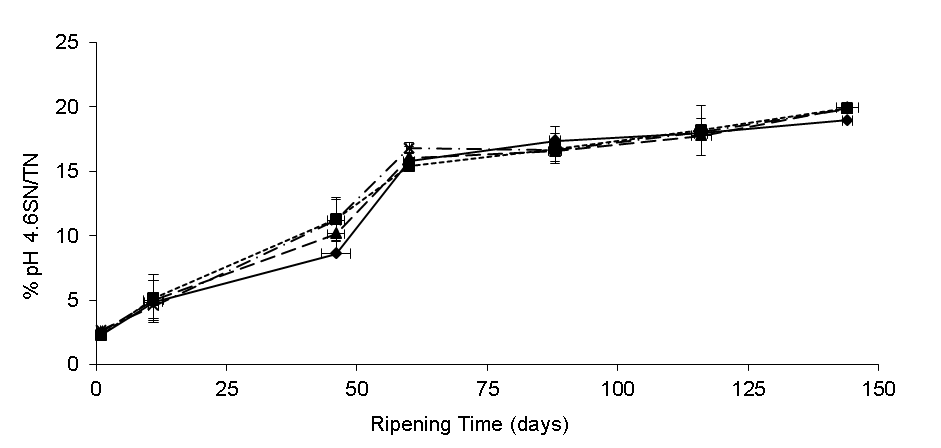


**Figure S5d:** The effect of different experimental set-up on cheese % pH4.6 soluble nitrogen over ripening time. Control cheese
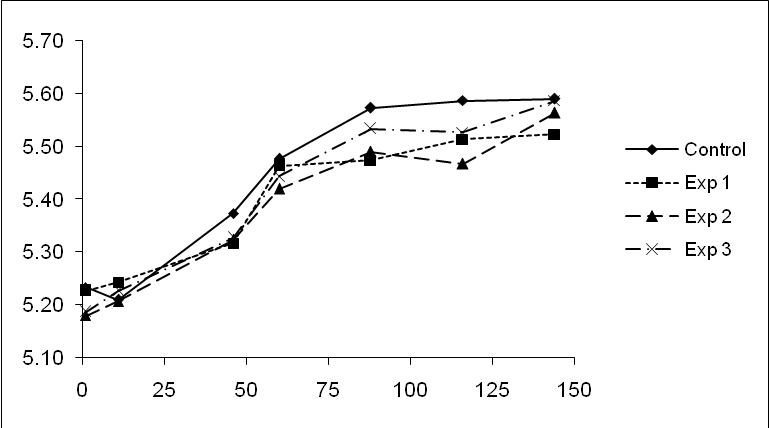
, experiment 1 cheese
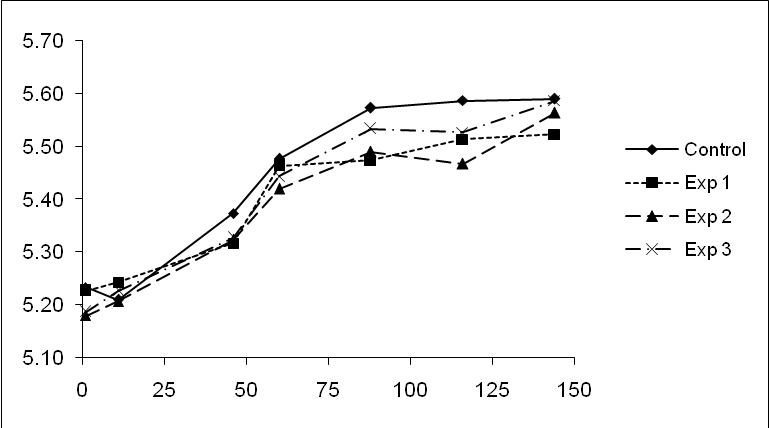
, experiment 2 cheese
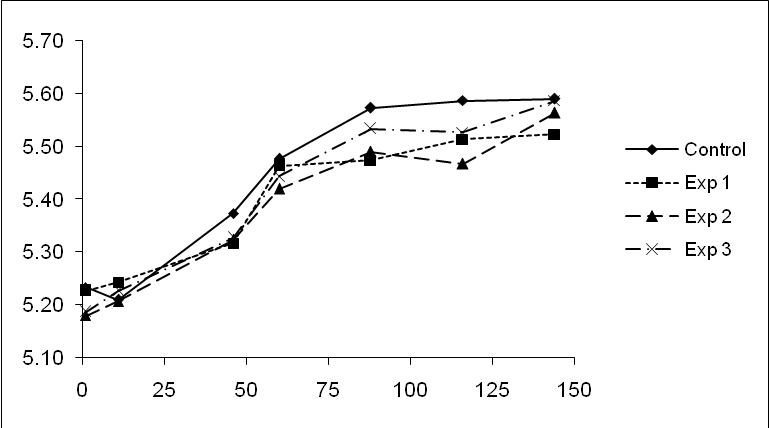
 and experiment 3 cheese
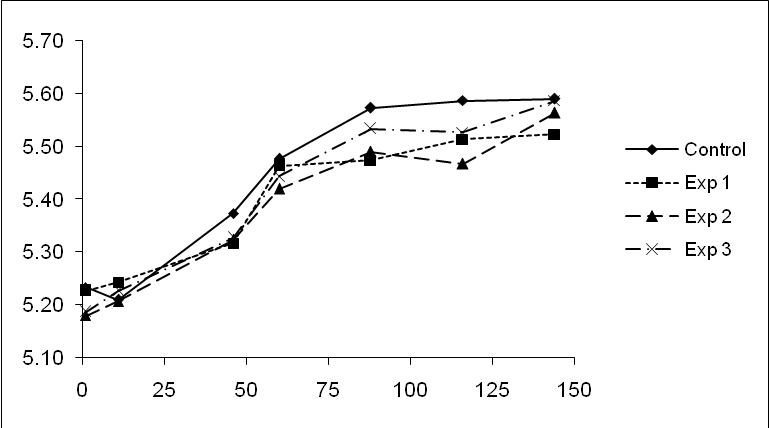
.


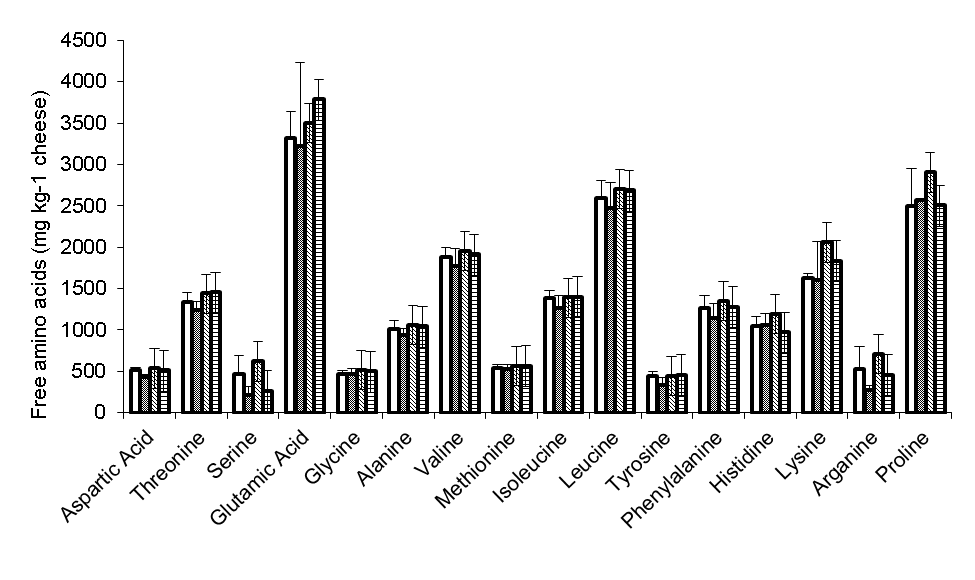


**Figure S5e**: The effect of different experimental set-up on free amino acid levels after 144 days ripening. Exp 1 ; Exp 2 ; Exp 3 ; Exp 4
